# Chlorogenic Acid as a Potential Therapeutic Agent for Cholangiocarcinoma

**DOI:** 10.3390/ph17060794

**Published:** 2024-06-17

**Authors:** Jiabao Liang, Tong Wen, Xiaojian Zhang, Xiaoling Luo

**Affiliations:** 1 Department of Experimental Research, Guangxi Medical University Cancer Hospital, Nanning 530021, China; liangjiabao1027@163.com (J.L.);; 2 Department of Immunology, School of Basic Medical Sciences, Guangxi Medical University, Nanning 530021, China

**Keywords:** AKR1B10, tumor immune microenvironment, chlorogenic acid, cholangiocarcinoma, apoptosis, macrophage polarization

## Abstract

Chlorogenic acid (CGA) has demonstrated anti-tumor effects across various cancers, but its role in cholangiocarcinoma (CCA) remains unclear. Our study revealed CGA’s potent anti-tumor effects on CCA, significantly suppressing cell proliferation, migration, colony formation, and invasion while inhibiting the epithelial–mesenchymal transition. CGA induced apoptosis, modulated cell cycle progression, and exhibited a stable binding affinity to AKR1B10 in CCA. AKR1B10 was highly expressed in RBE cells, and CGA treatment reduced AKR1B10 expression. Knocking out AKR1B10 inhibited the proliferation of RBE cells, whereas the overexpression of AKR1B10 promoted their proliferation. Additionally, CGA suppressed the proliferation of RBE cells with AKR1B10 overexpression. Mechanistically, AKR1B10 activated AKT, and CGA exerted its inhibitory effect by reducing AKR1B10 levels, thereby suppressing AKT activation. Furthermore, CGA facilitated the polarization of tumor-associated macrophages towards an anti-tumor phenotype and enhanced T-cell cytotoxicity. These findings underscore CGA’s potential as a promising therapeutic agent for CCA treatment.

## 1. Introduction

In recent years, the incidence of cholangiocarcinoma (CCA), a common primary malignant tumor, has increased significantly worldwide [1,2]. This rise in CCA cases has driven the need for improved treatment strategies. Traditional methods such as surgical resection, chemotherapy, radiation therapy, and immunotherapy have shown varying degrees of success over the past few decades [3,4]. Although there have been advances in 5 year relative survival rates, particularly for early-stage CCA patients, those with advanced stages still face poor treatment outcomes [4,5]. Therefore, there is an urgent need for new therapeutic agents to address this gap in CCA management.

Chlorogenic acid (CGA), a prominent polyphenolic compound found in various vegetables, fruits, and certain coffee beverages, has gained significant attention for its potential health benefits in recent years [6,7]. Studies have highlighted its positive effects on neuroprotection, cardiovascular health, gastrointestinal health, kidney protection, liver protection, and the regulation of lipid metabolism [8,9,10]. Moreover, CGA has shown potential in suppressing the growth of tumors across several types of cancer, including colon, breast, lung, oral squamous cell carcinoma, and chronic myeloid leukemia [11,12,13]. However, research on the potential anti-tumor effects of CGA on CCA has been limited.

Given that apoptosis is a critical process for maintaining normal tissue growth, targeting apoptotic pathways presents a promising therapeutic approach for various malignancies [14]. Previous studies have also demonstrated the apoptotic effects of CGA in the treatment of other tumors [11].

Aldo-Keto reductase family 1 member B10 (AKR1B10), also referred to as Aldose reductase-like protein-1 (ARL-1), is an essential component of the human aldo-keto reductase (AKR) superfamily [15]. Recent research suggests that AKR1B10 may play a crucial role in the initiation and progression of various cancers [16,17]. In hepatocellular carcinoma (HCC), an increased expression of AKR1B10 is associated with poor prognoses, indicating its potential role in promoting tumor survival [18]. Similarly, in individuals with breast cancer, elevated levels of AKR1B10 are linked to resistance to chemotherapy drugs [19]. These findings underscore AKR1B10’s potential significance in cancer development, prognosis, and treatment response. However, there is limited research on AKR1B10 in cholangiocarcinoma, and currently, there are no reported studies on its role as a therapeutic target. Therefore, in this study, we investigated the impact of CGA on AKR1B10 and its subsequent anti-tumor effects.

In recent years, there has been a resurgence in understanding the role of macrophages in cancer. Tumor-associated macrophages (TAMs) are the predominant population of innate immune cells within the tumor microenvironment (TME). Derived from circulating monocytes, TAMs serve a crucial function in both tumor development and progression [20,21,22,23]. Depending on the cytokine profile in the microenvironment, these macrophages can adopt different activation states, including the pro-inflammatory M1 phenotype and the anti-inflammatory M2 phenotype [23,24]. In non-malignant or regressing tumors, most TAMs exhibit an M1-like phenotype characterized by pro-inflammatory activity, which is involved in microbial killing and tumor resolution [25]. In contrast, malignant tumors often host TAMs with an M2 phenotype that promotes tumor growth through the secretion of proangiogenic factors and immunosuppressive cytokines [26]. It is well-known that inducing TAM differentiation from an M2 to an M1 phenotype can enhance anti-tumor immune responses while reducing tumor growth and metastasis [22]. However, the impact of CGA-treated macrophage polarization on CCA and its influence on the CCA immune microenvironment remain poorly understood.

This study aimed to investigate the potential anti-tumor effects of CGA in CCA. Our findings revealed that CGA induces apoptosis in CCA cells. Additionally, we observed that CGA promotes the polarization of TAMs towards an M1 phenotype and enhances T-cell cytotoxicity. These findings open up a promising avenue for further research into CGA as a potential therapeutic agent for CCA, addressing the pressing need for improved treatment options in the advanced stages of this disease.

## 2. Results

### 2.1. CGA Inhibits the Proliferation of Human RBE Cells and Affects Cell Cycle Progression

**Figure 1:**
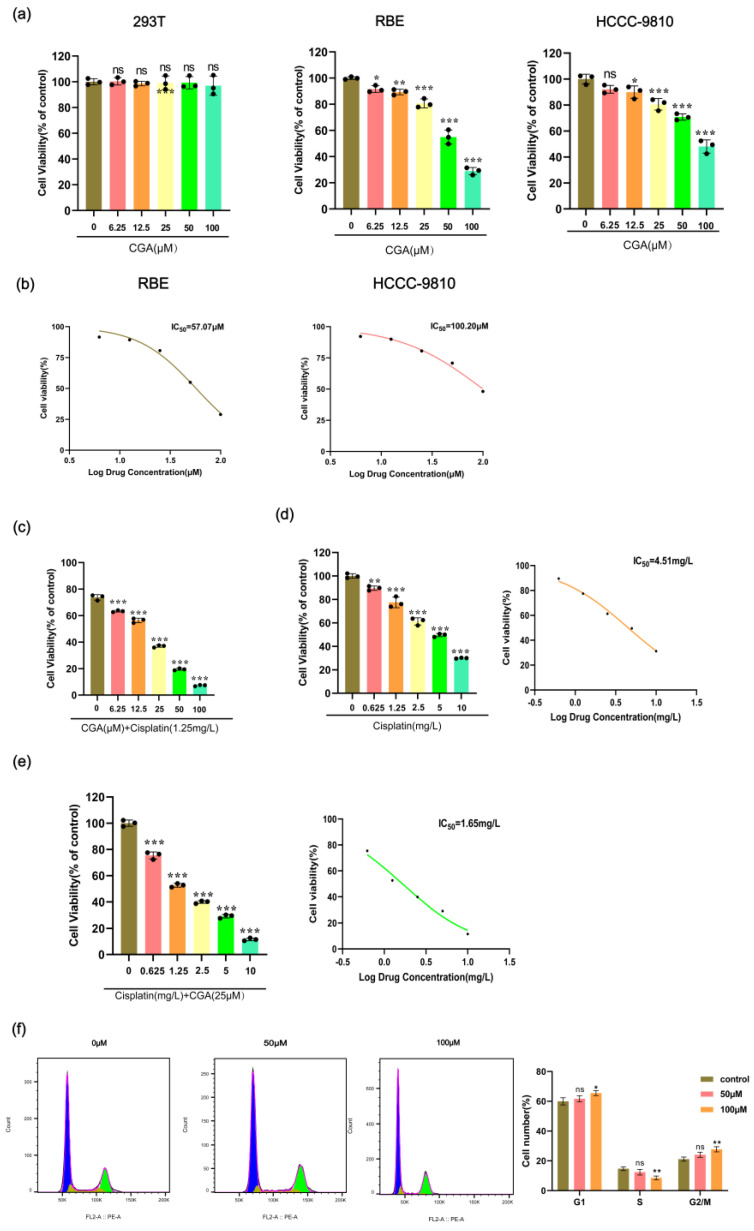
Effect of CGA on cell viability, proliferation, and cell cycle division of RBE cells. (**a**) 293T, RBE, and HCCC-9810 cell lines were incubated with CGA at concentrations ranging from 0 to 100 μM for 24 h. (**b**) IC_50_ curves of CGA in RBE cells and HCCC-9810 cells. (**c**) RBE cells were incubated for 24 h with a combination of 0–100 μM CGA and 1.25 mg/L cisplatin. (**d**) RBE cells were incubated for 24 h with cisplatin at concentrations ranging from 0 to 5 mg/L, and the corresponding IC_50_ curve was generated. (**e**) RBE cells were incubated for 24 h with cisplatin at concentrations ranging from 0 to 5 mg/L, along with 25 μM CGA, and the corresponding IC_50_ curve was generated. (**f**) RBE cells were exposed to 0 μM, 50 μM, and 100 μM CGA for 48 h, and cell cycle distribution was assessed using PI staining. The values represent the mean ± SD of three independent experiments. * *p* < 0.05, ** *p* < 0.01, *** *p* < 0.001, ns: no significance.

We conducted CCK-8 assays to assess the cytotoxic effects of chlorogenic acid (CGA) at various concentrations on 293T, RBE, and HCCC-9810 cell lines. The data suggested that, when CGA concentrations were varied from 0 to 100 μM, there was no significant impact on the viability of the 293T cells. However, as the CGA concentration increased, a dose-dependent reduction in cell viability was noted in both the RBE and HCCC-9810 cells (Figure 1a). Subsequent analysis revealed the IC_50_ values for CGA, with an IC_50_ of 57.07 μM for the RBE cells and 100.20 μM for the HCCC-9810 cells (Figure 1b). Given the lower IC_50_ value in the RBE cells, subsequent studies will focus on this cell line to further investigate the effects of CGA. At a cisplatin concentration of 1.25 mg/L, the viability of the RBE cells was approximately 73.76%. Co-administration with CGA at concentrations of 6.25, 12.5, 25, 50, and 100 μM resulted in a dose-dependent reduction in cell viability, with values decreasing to 63.34%, 56.39%, 36.89%, 19.46%, and 7.25%, respectively (Figure 1c). Furthermore, our observations indicated that the combined treatment of cisplatin with CGA (25 μM) yielded an IC_50_ of 1.65 mg/L, representing a notable decrease compared to the IC_50_ of 4.51 mg/L observed with cisplatin alone (Figure 1d,e). To investigate the anti-proliferative effects of CGA and its impact on cell cycle distribution, we performed cell cycle experiments. The results revealed a significant increase in the proportion of RBE cells in the G0/G1 phase, rising from 59.97% to 61.7% following treatment with 50 μM of CGA and further increasing to 65.5% after exposure to 100 μM of CGA (Figure 1f).

### 2.2. CGA Inhibits Migration, Epithelial–Mesenchymal Transition, Invasion, and Colony Formation of RBE Cells

**Figure 2:**
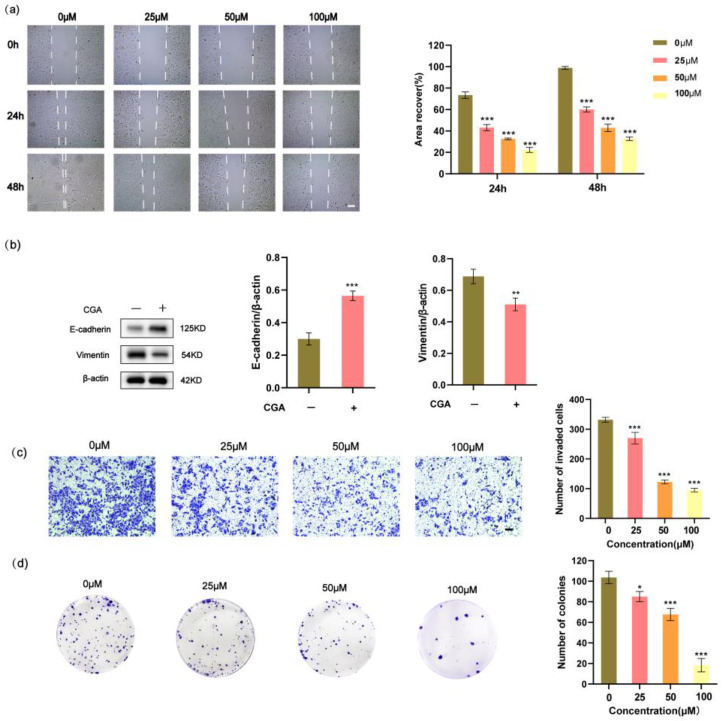
CGA inhibits the migration and invasion of RBE cells, along with suppressing their colony-forming ability and the expression of genes associated with EMT. (**a**) The effect of CGA on the migration of RBE cells using a wound healing assay. (**b**) Decreased expression of Vimentin and E-cadherin RBE cells following treatment with CGA as detected by Western blotting. (**c**) The effect of CGA on the migration of RBE cells using an invasion assay. (**d**) RBE cells were treated with varying concentrations of CGA (0, 25, 50, or 100 μM) for 48 h, followed by colony-forming assays. Scale bars, 50 µm. The values represent the mean ± SD of three independent experiments. * *p* < 0.05, ** *p* < 0.01, *** *p* < 0.001.

Cell migration plays an important role in cancer metastasis. To assess the migratory behavior of cancer cells, we conducted wound healing assays. The results revealed that RBE cells treated with CGA exhibited reduced migration distances compared to those without CGA treatment (Figure 2a). Vimentin and E-cadherin, crucial markers for EMT, showed distinct responses in RBE cells following CGA treatment. The vimentin levels significantly decreased, whereas the E-cadherin levels increased after CGA treatment (Figure 2b). Furthermore, we observed a direct correlation between increasing the CGA concentrations and a consequential decrease in the invasive ability of RBE cells (Figure 2c). To evaluate the impact of CGA on the colony-forming ability of RBE cells, we cultured these cells in the presence of varying concentrations of CGA (0, 25, 50, or 100 μM) for 48 h and subsequently assessed their ability to form colonies. Remarkably, an increase in the CGA concentration resulted in a significant inhibition of colony formation (Figure 2d).

### 2.3. CGA Promotes Mitochondrial Damage and Apoptosis in RBE Cells

**Figure 3:**
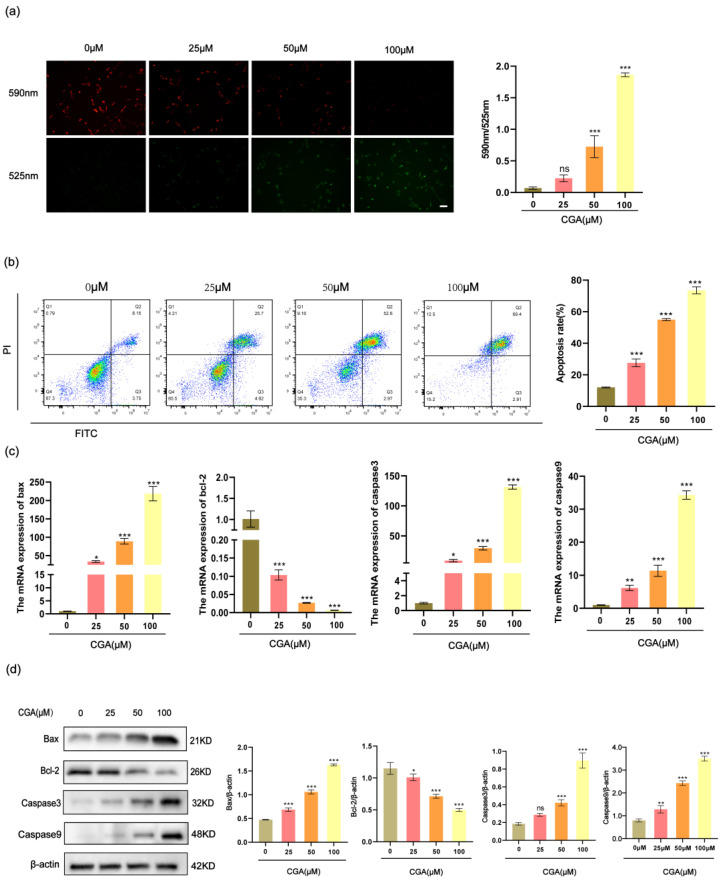
CGA induces mitochondrial damage and cell apoptosis in RBE cells. (**a**) JC-10 fluorescent probe was employed to evaluate MMP levels in RBE cells. (**b**) The apoptosis-inducing impact of CGA on RBE cells was analyzed through flow cytometry. (**c**) RT-qPCR was employed to assess mRNA expression levels of apoptosis-related factors, including caspase3, caspase9, bax, and bcl-2. (**d**) Western blotting was utilized to evaluate protein expression levels of apoptosis-related factors, such as caspase3, caspase9, bax, and bcl-2. The values represent the mean ± SD of three independent experiments. * *p* < 0.05, ** *p* < 0.01, and *** *p* < 0.001, ns: no significance.

MMP was evaluated with the JC-10 fluorescent probe, and the degree of mitochondrial depolarization was quantified by the red to green fluorescence intensity ratio. The RBE cells showed a notable reduction in MMP after being exposed to different concentrations of CGA for 24 h (Figure 3a). The dysregulation of apoptosis, or programmed cell death, plays a pivotal role in the development of various diseases, including cancer. In this assay, we used Annexin V and PI staining to observe the apoptosis rates of the CGA-treated RBE cells. The results indicated that the RBE cells treated with 0, 25, 50, or 100 μM CGA exhibited apoptosis rates of 12.01%, 27.55%, 54.95%, and 73.54%, respectively (Figure 3b). Additionally, we assessed the effects of CGA on the expression levels of several apoptosis-related genes in the RBE cells using RT-qPCR analysis and Western blotting. The results revealed that CGA significantly increased the expression levels of bax, caspase9, and caspase3 in the RBE cells while decreasing the expression level of bcl-2 (Figure 3c,d).

### 2.4. CGA Exerts Anti-Cholangiocarcinoma Effect through AKR1B10

**Figure 4:**
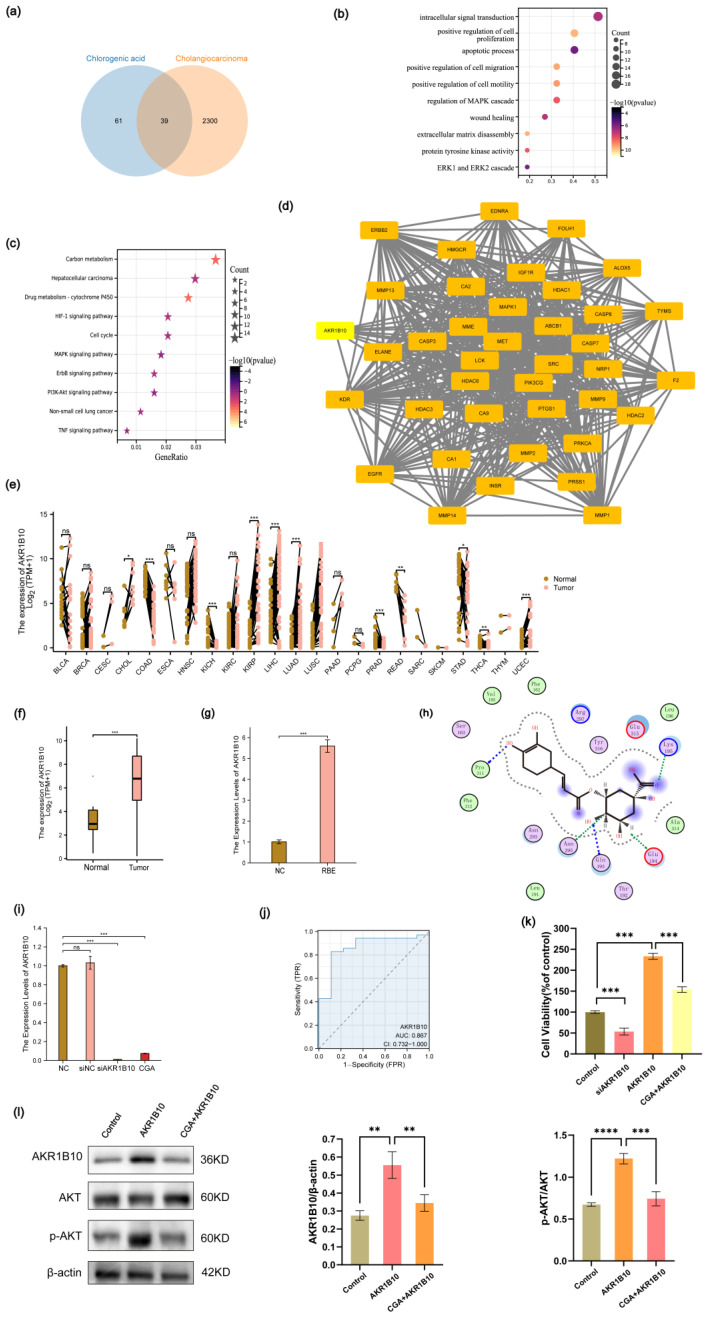
Elevated AKR1B10 Expression in various tumors, with emphasis on cholangiocarcinoma, and its interaction with chlorogenic acid. (**a**) The potential targets between CGA and CCA. (**b**) GO enrichment analysis. (**c**) KEGG pathway enrichment analysis. (**d**) PPI protein interaction. (**e**) AKR1B10 expression in different human cancers and normal tissues. (**f**) mRNA expression of AKR1B10 in normal tissues and CCA according to TCGA database. (**g**) mRNA expression of AKR1B10 in RBE cells. (**h**) Molecular docking results of chlorogenic acid and the protein of AKR1B10. (**i**) Effect of CGA on AKR1B10 mRNA expression. (**j**) The AUC curve of AKR1B10. (**k**) CCK-8 assay was used to examine the effects of siAKR1B10, AKR1B10 overexpression, and CGA combined with AKR1B10 overexpression on RBE cell proliferation. (**l**) Western blotting analysis of the effects of AKR1B10 overexpression and CGA combined with AKR1B10 overexpression on AKT protein phosphorylation in RBE cells. The values of (**g**,**i**,**k**,**l**) represent the mean ± SD of three independent experiments. * *p* < 0.05, ** *p* < 0.01, *** *p* < 0.001 and **** *p* < 0.0001, ns: no significance.

Through Swiss Target Prediction, we identified 100 target genes associated with CGA. Additionally, an analysis of CeneCards unveiled 2339 target genes related to CCA. Employing a Venn diagram, we illustrated 39 intersecting target genes common to both CGA and CCA (Figure 4a). A subsequent GO analysis revealed enrichment in processes such as intracellular signal transduction, the positive regulation of cell proliferation, apoptotic processes, the positive regulation of cell migration, the positive regulation of cell motility, the regulation of the MAPK cascade, wound healing, extracellular matrix disassembly, protein tyrosine kinase activity, and the ERK1 and ERK2 cascade (Figure 4b). Furthermore, KEGG analysis indicated enrichment in pathways associated with carbon metabolism, hepatocellular carcinoma, drug metabolism, the HIF-1 signaling pathway, the cell cycle, the MAPK signaling pathway, the ErbB signaling pathway, the PI3K-Akt signaling pathway, non-small cell lung cancer, and the TNF signaling pathway (Figure 4c). A protein–protein interaction (PPI) analysis pinpointed AKR1B10 as a significant target (Figure 4d). A pan-cancer analysis demonstrated elevated expression of AKR1B10 across various cancers, including CHOL, KIRP, LIHC, LUAD, LUSC, and UCEC (Figure 4e,f). Subsequent RT-qPCR validation confirmed a heightened AKR1B10 expression in the RBE cells (Figure 4g). For molecular docking studies, we employed the MOE software (version 2019.0102). Lower binding energies indicate better docking results. The top five docking results for CGA to AKR1B10 were −9.3 kcal/mol, −7.4 kcal/mol, −7.3 kcal/mol, −3.6 kcal/mol, and −2.0 kcal/mol. Docking figures presenting the binding sites, docking modes, and protein residues for these top-ranked interactions are displayed (Figure 4h and Supplement Figure S1). Subsequently, RT-qPCR analysis also revealed that CGA can inhibit the mRNA expression levels of AKR1B10 (Figure 4i). An analysis of TCGA-CHOL data revealed a notable area under the curve (AUC) value of 0.867 for AKR1B10 (Figure 4j). The CCK-8 assays demonstrated that the overexpression of AKR1B10 promoted the proliferation of RBE cells. Conversely, CGA was able to inhibit the proliferation of RBE cells overexpressing AKR1B10, while knockdown of AKR1B10 reduced the proliferation of RBE cells (Figure 4k). To investigate whether CGA regulates the biological functions of RBE cells by targeting AKR1B10 and affecting AKT, we performed a Western blot analysis. The results indicated that AKR1B10 overexpression increased AKT protein phosphorylation. Conversely, CGA treatment in RBE cells overexpressing AKR1B10 decreased both AKR1B10 protein expression and AKT protein phosphorylation (Figure 4l).

### 2.5. CGA Promotes the Polarization of M0 Macrophages to an M1 Phenotype, Which Consequently Increases the Cytotoxicity of T Lymphocytes

**Figure 5:**
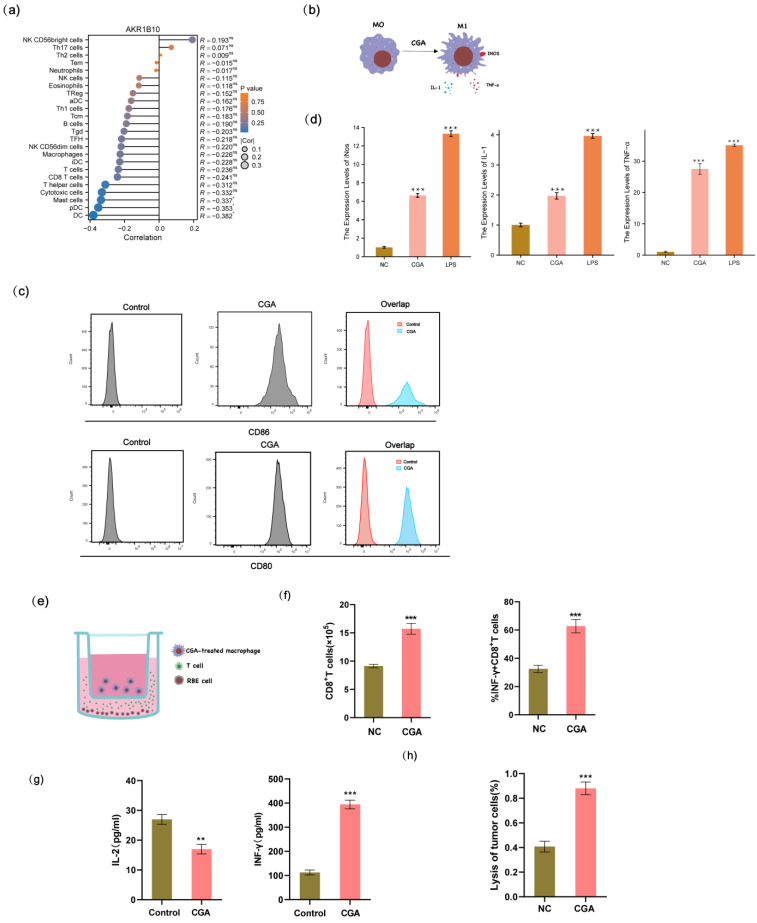
The polarization of macrophages and assessment of the cytotoxicity of CGA-treated macrophages against RBE cells, as well as the activation of T lymphocytes. (**a**) The infiltration of 24 immune cell types in TCGA-CHOL patients. (**b**) Schematic diagram of M0 macrophage polarization to M1 phenotype following CGA treatment. (**c**) The effect of CGA on macrophage polarization was detected by flow cytometry. (**d**) The relative mRNA expression levels of iNOS, TNF-α, and IL-1 were measured to assess the impact of CGA on macrophage polarization compared to the control group. (**e**) Schematic diagram of the co-culture of CGA-treated macrophages, T cells, and RBE cells. (**f**) the frequency of total CD8+ T cells and IFN-γ + CD8+ T cells upon stimulation with CGA. (**g**) Detection of T cell cytokines by ELISA. (**h**) LDH release assay to assess the cytotoxicity of T cells against RBE cells. The values of (**d**,**f**,**g**,**h**) represent the mean ± SD of three independent experiments. * *p* < 0.05, ** *p* < 0.01, and *** *p* < 0.001, ns: no significance.

The samples were stratified into distinct subgroups based on the median expression levels of AKR1B10. Subsequently, we employed the ssGSEA method to investigate the distribution of 24 types of immune cells within different subgroups in cholangiocarcinoma tissues sourced from the TCGA-CHOL dataset. The samples were categorized into distinct subgroups based on the median expression levels of AKR1B10. Subsequently, we utilized the ssGSEA method to explore the correlation between AKR1B10 expression and the distribution of 24 types of immune cells in the cholangiocarcinoma tissues obtained from the TCGA-CHOL dataset. The results indicated a negative association between the expression of AKR1B10 and several immune cell types, with statistically significant differences observed in cytotoxic cells, mast cells, dendritic cells (DCs), and plasmacytoid dendritic cells (pDCs) (Figure 5a). M1 macrophages are renowned for their potent anti-tumor activity. The expression of CD80 and CD86, which are recognized as markers for M1 macrophages, was significantly upregulated following CGA treatment (Figure 5c). Notably, treatment with CGA led to an increase in the expression levels of iNOS, TNF-α, and IL-1 in macrophages when compared to the negative control group (Figure 5d). Here, we employed flow cytometry to demonstrate that the CGA-treated macrophages induced the activation and expansion of CD8+ T cells, as well as an increase in the proportion of IFN-γ + CD8+ T cells (Figure 5f). Additionally, ELISA was performed to evaluate the levels of IFN-γ and IL-2, which serve as reliable indicators for assessing the cytotoxic activity of T lymphocytes (Figure 5g). Furthermore, we conducted the lactic acid dehydrogenase (LDH) release assay to gauge the extent to which cancer cells were targeted and eliminated by T lymphocytes (Figure 5h).

## 3. Materials and Methods

### 3.1. Cell Culture and Treatments

The RBE cells, HCCC-9810 cells, and 293T cells were supplied by the Cell Bank of Chinese Academy of Science (Shanghai, China) and cultured according to the provided instructions. Cultivation was carried out in either Dulbecco’s Modified Eagle Medium (DMEM) or Roswell Park Memorial Institute 1640 (RPMI-1640) medium, both supplemented with 10% fetal bovine serum (FBS), 100 U/mL of penicillin, and 100 mg/mL of streptomycin, at 37 °C in a humidified incubator containing 5% CO_2_. DMEM, RPMI-1640, FBS, penicillin, and streptomycin were all purchased from Gibco, Carlsbad, CA, USA. Chlorogenic acid (CGA) was purchased from Aladdin (C109402, Shanghai, China), dissolved in DMSO, and added to the medium to achieve the desired final concentration. An equal volume of DMSO was used as a negative control. To knock down AKR1B10 expression, AKR1B10-specific small interfering RNA (siAKR1B10) (5′-GGCCTATGTCTATCAGAATGAAC-3′) or a nonspecific siRNA control (siNC) (5′-AAGACAUUGUGUGUCCGCCTT-3′) were used to transfect the RBE cells. For AKR1B10 overexpression, the RBE cells were transfected with pcDNA3.1-AKR1B10, using an empty vector as a control. According to the manufacturer’s guidelines, we conducted transfections with Lipofectamine 2000 (11668019, Invitrogen, Carlsbad, CA, USA). In brief, one day before transfection, 5 × 10^5^ cells per well were seeded in antibiotic-free medium. The cells were ensured to be 60–70% confluent at the time of transfection. The siRNA or plasmid was diluted in Opti-MEM serum-free medium, mixed gently, and incubated at room temperature for 5 min. Lipofectamine 2000 was gently mixed before use and diluted in Opti-MEM. The diluted siRNA or plasmid was combined with Lipofectamine 2000, mixed gently, and incubated at room temperature for 20 min. The transfection mixture was added to the cells, gently mixed, and incubated at 37 °C in a CO_2_ incubator. After 6 h, the medium was replaced with complete medium and with experiments were proceeded with as needed.

### 3.2. Cell Viability Assay

The 293T, RBE, and HCCC-9810 cells were seeded at a density of 1 × 10^4^ cells per well in 96-well plates and incubated overnight. The following day, different concentrations of CGA (0, 6.25, 12.5, 25, 50, and 100 μM) were administered to the cells. After a 24 h incubation period, cell viability was assessed using the Cell Counting Kit-8 (CCK-8) assay, following the manufacturer’s instructions (C0037, Beyotime Biotechnology, Shanghai, China).

### 3.3. Colony Formation Assay

Following treatment with CGA at concentrations of 0, 25, 50, and 100 μM, the cells were incubated for 48 h, then trypsinized and plated in 6-well plates at around 500 cells per well. After a two-week incubation period, the cells were fixed using 4% paraformaldehyde for 15–30 min and stained with 0.5% crystal violet for 10–20 min. The colony counts were subsequently quantified.

### 3.4. Evaluation of Cell Cycle by PI-Staining Assay

Flow cytometry was used to evaluate the cell cycle distribution. The cells were fixed in 70% ice-cold ethanol and incubated overnight at 4 °C. After centrifugation at approximately 1000× *g* for 3–5 min to precipitate the cells, they were washed twice with ice-cold PBS and then resuspended in 1 mL of PI staining solution (C1052, Beyotime Biotechnology, Shanghai, China). After 10 min incubation in the dark at room temperature, the cell cycle phases (G0/G1, S, and G2/M) were analyzed using a flow cytometer (Beckman, Brea, CA, USA).

### 3.5. Wound Healing and Transwell Invasion Assay

The RBE cells were seeded in a six-well plate. Upon reaching confluence, a scratch was made in the cell monolayer. CGA was then added to the wells at concentrations of 0, 25, 50, and 100 µM. The width of the scratch was observed under a microscope at 0, 24, and 48 h. The culture medium in the lower chamber was supplemented with 10% FBS and had a volume of 600 μL. A single-cell suspension of 1 × 10^5^ cells was seeded into the upper chamber for migration assessment and on Matrigel (356234, Corning, Corning, New York, USA) for invasion evaluation. Following 24 h of incubation, the cells in the upper chamber were fixed in 4% paraformaldehyde for 5 min and stained with 0.1% crystal violet for another 5 min, followed by two PBS washes. The migrated cells on the membrane were then quantified using microscopic examination.

### 3.6. Mitochondrial Membrane Potential (MMP) JC-10 Assay

MMP was assessed using the JC-10 assay kit from Solarbio (CA1310, Beijing, China). RBE cells, seeded at a density of 2 × 10^5^ cells per mL in 12-well plates, were treated with varying concentrations of CGA (0, 25, 50, and 100 μM) for 24 h. Subsequently, the medium was replaced with fresh medium, and JC-10 dye was added. The cells were incubated in a 37 °C incubator for 20 min. After incubation, the cells were washed with JC-10 staining buffer. The JC-10 aggregates were visualized using a fluorescence microscope at 525 nm and 590 nm, and fluorescence intensity was quantified using ImageJ software (version 1.54). Changes in mitochondrial membrane potential were determined by calculating the ratio of JC-10 aggregates (Em 525 nm) to monomers (Em 590 nm).

### 3.7. Evaluation of Apoptosis by Annexin V/PI Staining Assay

Cells were treated with 0, 25, 50, or 100 μM of CGA for 48 h. Subsequently, the cells were collected and stained with Annexin V-FITC and PI (C1062M, Beyotime Biotechnology, Shanghai, China). The procedure was as follows: the culture medium was transferred to a centrifuge tube, and the adherent cells were washed with PBS. The cells were digested with trypsin without EDTA. The previously collected medium was added back, and the cells were gently pipetted off and transferred to the centrifuge tube. After centrifugation at 1000× *g* for 5 min, the supernatant was discarded and the cell pellet was resuspended in 195 μL of Annexin V-FITC binding buffer. Next, 5 μL of Annexin V-FITC and 10 μL of PI staining solution were added and thoroughly mixed. The cells were then incubated at room temperature in the dark for 10 min. Fluorescence detection was carried out using a flow cytometer, and the apoptosis rates were subsequently analyzed with Flow Jo software (version 10.8.1).

### 3.8. Real-Time Quantitative PCR Analysis

Using a total RNA kit (R6834, Omega, Norcross, GA, USA), the total RNA was extracted from the cells. Cell lysis was carried out using lysis buffer, and the supernatant was then transferred to a filter column for centrifugation. Ethanol was then added to the binding column. The RNA was centrifuged and absorbed, followed by three washing steps and drying. Finally, DEPC water was added to elute the RNA, and the RNA concentration was measured. The RNA was then reverse transcribed using a reverse transcription kit (M1631, Thermo Fisher Scientific, Waltham, MA, USA). The total RNA extracted was mixed with RT Buffer Mix, RT Enzyme Mix, and water according to the instructions. The reaction was then incubated at 42 °C for 30 min to reverse transcribe into cDNA. SYBR Green Real-Time PCR Master Mix (11762100, Thermo Fisher Scientific, Waltham, MA, USA) was utilized for forty cycles of PCR in the Roche Light Cycler 480 system. The real-time PCR primers employed were as follows (Table 1). Fluorescence-quantitative PCR was performed using a 20 μL reaction system, and the 2^−ΔΔCt^ method was used to quantify the relative expression levels.

### 3.9. Western Blotting

Cell-derived protein samples were prepared using RIPA buffer (P0013B, Beyotime, Shanghai, China) supplemented with a cocktail inhibitor (11697498001, Roche, Munich, Germany) and subjected to a 15 min boiling process. Subsequently, 15–30 μg of each sample was loaded onto an SDS-polyacrylamide gel. After SDS-PAGE, proteins were transferred from the gel to a polyvinylidene fluoride (PVDF) membrane (IPVH00010, Millipore, Darmstadt, Germany). Following a 2 h blocking step with 5% fat-free milk or bovine serum albumin (for phosphorylated proteins) at room temperature, the membrane was incubated overnight at 4 °C with primary antibodies. The antibodies used were E-cadherin (340341, 1:1000, Zenbio, Chengdu, China), Vimentin (R22775, 1:1000, Zenbio, Chengdu, China), Bax (CY5059, 1:1000, Abways, Shanghai, China), Bcl-2 (CY5032, 1:500, Abways, Shanghai, China), Caspase3 (CY5051, 1:500, Abways, Shanghai, China), Caspase9 (CY5049, 1:500, Abways, Shanghai, China), AKR1B10 (ab192865, 1:1000, Abcam, Cambridge, UK), AKT (9272, 1:1000, CST, Beverly, MA, USA), p-AKT (4060, 1:2000, CST, Beverly, MA, USA), and β-actin (AB0035, 1:5000, Abways, Shanghai, China). After washing with TBS-T, secondary antibodies (RS0002, ImmunoWay, Shanghai, China) were applied for 1 h. The target proteins were then visualized using an ECL Western Blotting substrate and detected with an Odyssey Imaging System (Li-COR, Lincoln, NE, USA).

### 3.10. Prediction of Target Genes in Chlorogenic Acid and Cholangiocarcinoma

The identification of the chlorogenic acid target genes involved using the Swiss Target Prediction database (http://www.swisstargetprediction.ch/, accessed on 20 June 2023) and the SuperPred database (https://prediction.charite.de/index.php, accessed on 20 June 2023). To maintain relevance to human biology, the analysis was restricted to genes found in Homo sapiens, and duplicate entries were removed to create a refined list. Simultaneously, the exploration for target genes linked to cholangiocarcinoma utilized GeneCards (http://www.genecards.org/, accessed on 21 June 2023). The search employed the keyword “Cholangiocarcinoma” to ensure precision in retrieving relevant data. The results were then integrated into the overall analysis.

### 3.11. Construction of Venn Diagram and the Protein-Protein Interaction (PPI) Net Work

The investigation into the potential target genes associated with both chlorogenic acid and cholangiocarcinoma was conducted using InteractiVenn (http://www.interactivenn.net/, accessed on 23 June 2023). The primary objective was to identify the specific target genes of chlorogenic acid that are connected to cholangiocarcinoma. The findings were visually represented through a Venn diagram, highlighting the common potential target genes overlapping between chlorogenic acid and cholangiocarcinoma. Additionally, the intersection of these target genes was further analyzed using a protein–protein interaction network via the STRING database version 11.5 (https://string-db.org/, accessed on 24 June 2023).

### 3.12. Molecular Docking Validation of the Binding Capacity between Chlorogenic Acid and AKR1B10

The investigation employed a molecular docking approach to analyze the interactions between chlorogenic acid and AKR1B10. The three-dimensional structure of chlorogenic acid was retrieved from the PubChem database and converted into Mol2 format using Open Babel software (version 3.1.1). The template structure of the target protein AKR1B10 (Protein ID: O60218) was obtained from the UniProt database. The molecular docking of chlorogenic acid with AKR1B10 was then performed using the MOE software (version 2019.0102).

### 3.13. Gene Ontology and Pathway Enrichment Analysis

We conducted a thorough investigation into the biological mechanisms underlying the synergistic effects of chlorogenic acid against cholangiocarcinoma. To identify the molecular pathways affected by chlorogenic acid and its potential effects on cholangiocarcinoma, Gene Ontology (GO) and Kyoto Encyclopedia of Genes and Genomes (KEGG) pathway enrichment analyses were conducted. Subsequently, we submitted the therapeutic targets of chlorogenic acid against cholangiocarcinoma to the Sangerbox database (http://vip.sangerbox.com/home.html, accessed on 15 July 2023) for further analysis and exploration. The results highlighted significant findings from both the GO and KEGG analyses.

### 3.14. AKR1B10 Expression in Pan-Cancer and Immune Infiltration Analysis

We examined the mRNA expression of AKR1B10 across diverse cancer types using the TCGA database (https://portal.gdc.cancer.gov, accessed on 1 June 2023). RNAseq data in TPM format were downloaded from the TCGA project, encompassing 33 distinct tumor types. Statistical analysis was conducted using the stats and car R packages, ensuring compliance with prerequisites before analysis. Data from the TCGA-CHOL (cholangiocarcinoma) project were also acquired from the same database, including STAR-processed RNAseq data in FPKM format. Further analysis involved assessing the correlation between the key variables and immune infiltration matrix data, with the results visually presented using ggplot2.

### 3.15. Polarization of Macrophages In Vitro

THP-1 cells were also supplied by the Cell Bank of the Chinese Academy of Sciences (Shanghai, China) and cultured according to the provided instructions. The THP-1 cells were induced with phorbol esters, seeded at a density of 5 × 10^5^ M0 macrophages per well in a 6-well plate, and treated with 50 μM of CGA for 24 h. Macrophages stimulated with LPS (IL2020, Solarbio, Beijing, China) served as positive controls. The expression levels of iNOS, IL-1, and TNF-α were assessed using RT-qPCR. Flow cytometry was employed to determine the expression levels of CD80 and CD86 on macrophages.

### 3.16. Isolation and Purification of T Cells

Using Ficoll density gradient centrifugation (P8680, Solarbio, Shanghai, China), peripheral blood mononuclear cells (PBMCs) were isolated from 20 mL of venous blood. CD3+ T cells were purified using a CD3+ T cell separation reagent kit (17751, StemCell Technologies, Vancouver, BC, Canada). This process leverages the ability of cell surface antigens to bind to magnetic beads coated with specific antibodies. Under an external magnetic field, the cells were selectively captured by the antibodies on the magnetic beads, allowing for the isolation of CD3+ T cells. The T cells were then activated by adding CD3/CD28 T cell activation beads (422603, BioLegend, San Diego, CA, USA) at a 1:1 ratio to the number of CD3+ T cells and cultured in IL-2-supplemented RPMI-1640 culture medium.

### 3.17. Cytotoxicity of T Cells

The RBE cells were placed in 96-well plates at a seeding density of 1 × 10^5^ cells per well and allowed to incubate for 12 h. Subsequently, CGA-treated macrophages (1 × 10^5^ cells) were introduced into the upper chamber of a co-culture system with a pore size of 1 μm, and T cells (5 × 10^5^ cells) were added to the lower chamber containing the pre-seeded RBE cells. M0 macrophages that had not been treated with CGA served as the control group. The samples were then centrifuged and the supernatant was collected. The supernatant was analyzed using an LDH cytotoxicity assay kit (C0016, Beyotime, Shanghai, China). The cytotoxicity was calculated as follows: Cytotoxicity (%) = (Sample Absorbance − Control Absorbance)/(Maximum Enzyme Activity Absorbance − Control Absorbance) × 100. Additionally, flow cytometry was used to quantify the population of the CD8+ T cells and determine the proportion of IFN+ CD8+ T cells.

### 3.18. ELISA for the Analysis of Secreted Cytokines

The concentrations of cytokines (IFN-γ and IL-2) were determined using ELISA kits (IL-2: SEKH-0008, IFN-γ: SEKH-0046, Solarbio, Shanghai, China), following the manufacturer’s instructions. After 24 h of co-culture, cell culture media from different treatment groups were collected, appropriately diluted, and analyzed using a microplate reader.

### 3.19. Statistical Analysis

The results were presented as the Mean ± SD from three independent biological replicates. Differences among three or more groups were analyzed using ANOVA, while comparisons between two groups were conducted using the Student’s *t*-test. Statistical significance was determined with a *p*-value of less than 0.05.

## 4. Discussion

Cholangiocarcinoma is a prevalent malignant tumor that primarily affects the bile ducts in adults, and it poses a significant global health concern due to its high mortality and morbidity rates [27]. While platinum-based chemotherapy has traditionally been the mainstay of systemic treatment for advanced CCA, it is frequently associated with the development of drug resistance, leading to poor clinical outcomes [2,28]. Therefore, there is a pressing need for the development of new agents that not only exhibit a low toxicity, but are also effective against advanced CCA. In this context, naturally occurring plant-derived compounds with anticancer properties have emerged as promising candidates for cancer prevention and treatment.

CGA is a well-known antibacterial, antiviral, and anti-inflammatory agent [10,29]. Furthermore, there is a growing body of evidence suggesting its potential as an anticancer agent. In this study, CGA was found to inhibit the proliferation, growth, viability, and colony-forming ability of RBE cells. These findings align with a recent study that demonstrated CGA’s significant inhibition of the proliferation of the lung cancer cell line A549 by targeting annexin A2. This interaction between annexin A2 and actin may inhibit the progression and migration of tumor cells [12]. DDP serves as a primary treatment for CCA, yet, its effectiveness is hindered by the widespread use of high doses and repeated treatment cycles in clinical oncology, resulting in undesirable side effects [30]. This study revealed that CGA effectively amplified the anti-viability effects of DDP on RBE cells. The combined treatment exhibited a significantly superior efficacy compared to DDP alone, with the combined groups necessitating lower IC_50_ concentrations than the DDP-alone treatment group. These findings suggest that CGA has the potential to enhance the therapeutic effects of DDP against cholangiocarcinoma, thereby potentially alleviating the side effects associated with DDP treatment. Moreover, our findings revealed that CGA induced cell cycle arrest in the G0/G1 phase, further highlighting its potential as a multifaceted anticancer agent.

In a study conducted by Hsu PH et al., it was demonstrated that CGA induced significant cell death in MCF-7 breast cancer cells [31]. Another study by Bandyopadhyay et al. suggested that CGA increased the apoptotic rate of chronic myelogenous leukemia cells by modulating p38 mitogen-activated protein kinase [32]. The activation of the caspase cascade predominantly occurs via the mitochondrial pathway, a process intricately regulated by the dynamic interplay between pro-apoptotic proteins such as bax and anti-apoptotic proteins such as bcl-2 [33]. These proteins, integral members of the bcl-2 protein family, govern the intrinsic apoptosis pathway. Bax acts as a pro-apoptotic factor by increasing mitochondrial membrane permeability. This leads to the release of cytochrome c from the mitochondria into the cytoplasm, which then triggers the ensuing caspase cascade reaction. Conversely, the anti-apoptotic protein bcl-2 impedes the release of cytochrome c from mitochondria by binding to the bax protein [34]. The susceptibility of cells to apoptotic stimuli is intricately determined by the delicate balance of these regulatory proteins. In our study, we noted a reduction in the mitochondrial membrane potential in the RBE cells upon exposure to CGA, indicating its potential to induce mitochondrial dysfunction. Furthermore, we found that CGA induced an upregulation in bax expression while simultaneously causing a downregulation in bcl-2 expression. In this study, we noted that exposure to CGA primarily triggered apoptosis in the RBE cells and changes in MMP, as substantiated by elevated levels of caspase3, caspase9, and bax, along with decreased levels of bcl-2.

Caspases, pivotal mediators of apoptosis, induce cell death by cleaving various intracellular substrates, ultimately resulting in cell lysis [35]. Caspase9, an initiator caspase in the cascade of cysteine aspartyl proteases, becomes activated when cytochrome c released into the cytoplasm binds and activates Apaf-1 [36]. Once activated, caspase 9 initiates the activation of effector caspase3, which then triggers the activation of the downstream target PARP, ultimately leading to cell apoptosis [37]. In our investigation, we observed an upregulation of caspase 3 and caspase 9 expression in the RBE cells following CGA treatment. Consequently, CGA could potentiate apoptosis in the RBE cells through the mitochondria-mediated caspase-dependent pathway.

We subsequently delved into the potential targets of CGA in its role against CCA. An analysis of the TCGA database indicated an overexpression of AKR1B10 in various tumor cells, with a notably high expression in cholangiocarcinoma. This upregulation of AKR1B10 mRNA levels in RBE cells was further validated by RT-qPCR analysis.

Our network pharmacology research revealed that CGA interacted with multiple targets in CCA. According to the GO and KEGG pathway analyses, these targets were significantly enriched in signaling pathways that are crucial for tumor development and progression. Among these, AKR1B10 was identified as a principal target. Intrigued by the possibility of CGA binding to AKR1B10, we utilized molecular docking techniques and ascertained that CGA could form a stable complex with the AKR1B10 protein. At the same time, we found that CGA could reduce the expression of AKR1B10 in the RBE cells and reverse the excessive proliferation caused by AKR1B10 overexpression in the RBE cells. These findings imply that AKR1B10 could be a key therapeutic target for CGA in the management of CCA. AKT, a serine/threonine kinase, is critical in mediating multiple signal transduction pathways and plays key roles in cell proliferation, migration, and apoptosis [38]. The expression of AKT is dysregulated in various malignancies, and its activation can promote cholangiocarcinoma tumorigenesis [39]. Multiple upstream signaling factors can activate AKT. Jiayao Qu et al. discovered that AKR1B10 enhances breast cancer cell proliferation and migration through the PI3K/AKT/NF-κB signaling pathway [40]. In our study, we found that the overexpression of AKR1B10 induced AKT activation in the RBE cells. Conversely, in CGA-treated RBE cells, the overexpressed AKR1B10 was inhibited, leading to a subsequent decrease in AKT expression.

An analysis of immune infiltration revealed a negative association between AKR1B10 expression and several immune cell types, particularly in cytotoxic cells, mast cells, dendritic cells (DCs), and plasmacytoid dendritic cells (pDCs). This suggests that CGA may exert anti-tumor effects by influencing the immune microenvironment. The tumor microenvironment (TME) plays a crucial role in cancer initiation and progression, with tumor-associated macrophages (TAMs) prominently implicated in promoting tumorigenesis and metastasis [41]. Targeting TAMs has emerged as an important approach in tumor therapy research. It encompasses key aspects such as inhibiting TAM recruitment within the TME, suppressing mechanisms that sustain TAM survival, and enhancing TAM polarization toward the M1 phenotype, which is known for its potent anti-tumor activity while attenuating TAM-induced tumor angiogenesis [20]. The increase in M1-type macrophage polarization stands as a crucial anti-tumor mechanism. Our findings indicated that CGA promoted M0 macrophage polarization into the M1 phenotype, aligning with previous studies on CGA as an immunomodulator with anti-tumor properties that shift TAMs from the M2 to the M1 phenotype. This modulation within the TME inhibited glioblastoma growth. Additionally, we observed that CGA-treated macrophages significantly enhanced T cell-mediated cytotoxicity, thereby augmenting their anti-tumor efficacy against CCA.

However, it is important to acknowledge that the antiproliferative effects of CGA may also involve molecular mechanisms other than apoptosis. Therefore, further investigation is warranted to comprehensively understand the mechanisms underlying CGA’s anticancer effects. Additionally, the role of AKR1B10 in CCA and its specific interaction with CGA necessitate further validation to provide a more complete understanding of the potential therapeutic pathways involved.

## 5. Conclusions

In summary, CGA exhibits substantial anti-tumor effects in the treatment of CCA. It achieves this by inhibiting cell proliferation, reducing colony formation, suppressing migration and invasion, inducing apoptosis, and altering the cell cycle distribution in CCA cells. CGA may exert its anti-cholangiocarcinoma effects by targeting AKR1B10 and subsequently influencing AKT. Additionally, CGA can target AKR1B10 and promote the polarization of TAMs towards an anti-tumor phenotype, enhancing T-cell cytotoxicity. These findings collectively underscore the significant therapeutic potential of CGA for CCA.

## Figures and Tables

**Table 1:** Primer sequences.

Genes	Forward Primer	Reverse Primer
Caspase-3	ATGGAAGCGAATCAATGGACTCTGG	TGAGGTTTGCTGCATCGACATCTG
Caspase-9	GACCAGAGATTCGCAAACCAGAGG	CGGACTCACGGCAGAAGTTCAC
Bax	GATGCGTCCACCAAGAAGCTGAG	CACGGCGGCAATCATCCTCTG
Bcl-2	GTGTGTGGAGAGCGTCAACCG	ACAGCCAGGAGAAATCAAACAGAGG
iNos	CGTGCGTTACTCCACCAACAATG	GGTAGCCAGCATAGCGGATGAG
TNF-α	AATGGCGTGGAGCTGAGAGATAAC	CGGCTGATGGTGTGGGTGAGG
AKR1B10	AAGTCTCCTCTTGGCAAAGTGAAAG	CTTGGATCTTCTCTTGGATGGCTTC
β-actin	CCTGGACTTCGAGCAAGAGATGG	CAGGAAGGAAGGCTGGAAGAGTG

## Data Availability

Due to our institution’s data security policy, the data are not publicly available but can be requested from the corresponding author.

## Supplementary Materials

The following supporting information can be downloaded at: https://www.mdpi.com/article/10.3390/ph17060794/s1, Figure S1: Molecular docking results of chlorogenic acid and the protein of AKR1B10.

## Abbreviations

*CGA*	Chlorogenic acid.
*CCA*	Cholangiocarcinoma.
*TAM*	Tumor-associated macrophage.
*TME*	Tumor microenvironment.
*iNOS*	Inducible nitric oxide synthase.
*AKR1B10*	Aldo-keto reductase family 1 member B10.
*CCA*	Cholangiocarcinoma.
*GO*	Gene Ontology.
*KEGG:*	Kyoto Encyclopedia of Genes and Genomes.
*Bax*	Bcl-2-associated X protein.
*Bcl-2*	B-cell lymphoma-2.
*EMT*	Epithelial–mesenchymal transition.
*AKR1B10*	Aldo-Keto reductase family 1 member B10.
*MMP*	Mitochondrial membrane potential.

## References

[1] Chen S., Chen Z., Li Z., Li S., Wen Z., Cao L., Chen Y., Xue P., Li H., Zhang D. (2022). Tumor-associated macrophages promote cholangiocarcinoma progression via exosomal Circ_0020256. Cell Death Dis..

[2] El-Diwany R., Pawlik T.M., Ejaz A. (2019). Intrahepatic Cholangiocarcinoma. Surg. Oncol. Clin. N. Am..

[3] Crane C.H., Koay E.J. (2016). Solutions that enable ablative radiotherapy for large liver tumors: Fractionated dose painting, simultaneous integrated protection, motion management, and computed tomography image guidance. Cancer.

[4] Greten T.F., Schwabe R., Bardeesy N., Ma L., Goyal L., Kelley R.K., Wang X.W. (2023). Immunology and immunotherapy of cholangiocarcinoma. Nat. Rev. Gastroenterol. Hepatol..

[5] Zhang K., Yu J., Yu X., Han Z., Cheng Z., Liu F., Liang P. (2018). Clinical and survival outcomes of percutaneous microwave ablation for intrahepatic cholangiocarcinoma. Int. J. Hyperth. Off. J. Eur. Soc. Hyperthermic Oncol. N. Am. Hyperth. Group.

[6] Plazas M., Prohens J., Cuñat A.N., Vilanova S., Gramazio P., Herraiz F.J., Andújar I. (2014). Reducing capacity, chlorogenic acid content and biological activity in a collection of scarlet (*Solanum aethiopicum*) and Gboma (*S. macrocarpon*) eggplants. Int. J. Mol. Sci..

[7] Naveed M., Hejazi V., Abbas M., Kamboh A.A., Khan G.J., Shumzaid M., Ahmad F., Babazadeh D., FangFang X., Modarresi-Ghazani F. (2018). Chlorogenic acid (CGA): A pharmacological review and call for further research. Biomed. Pharmacother..

[8] Bao L., Li J., Zha D., Zhang L., Gao P., Yao T., Wu X. (2018). Chlorogenic acid prevents diabetic nephropathy by inhibiting oxidative stress and inflammation through modulation of the Nrf2/HO-1 and NF-ĸB pathways. Int. Immunopharmacol..

[9] Bhandarkar N.S., Brown L., Panchal S.K. (2019). Chlorogenic acid attenuates high-carbohydrate, high-fat diet-induced cardiovascular, liver, and metabolic changes in rats. Nutr. Res..

[10] Zhou X., Zhang B., Zhao X., Lin Y., Wang J., Wang X., Hu N., Wang S. (2021). Chlorogenic acid supplementation ameliorates hyperuricemia, relieves renal inflammation, and modulates intestinal homeostasis. Food Funct..

[11] Changizi Z., Moslehi A., Rohani A.H., Eidi A. (2021). Chlorogenic acid induces 4T1 breast cancer tumor’s apoptosis via p53, Bax, Bcl-2, and caspase-3 signaling pathways in BALB/c mice. J. Biochem. Mol. Toxicol..

[12] Wang L., Du H., Chen P. (2020). Chlorogenic acid inhibits the proliferation of human lung cancer A549 cell lines by targeting annexin A2 in vitro and in vivo. Biomed. Pharmacother..

[13] Sharma G., Kamboj M., Narwal A., Bhardwaj R., Yadav P. (2022). Cytotoxic Role of Chlorogenic Acid on Oral Squamous Cell Carcinoma Cell Line. Indian J. Otolaryngol. Head Neck Surg. Off. Publ. Assoc. Otolaryngol. India.

[14] Liu T., Wu Z., He Y., Xiao Y., Xia C. (2020). Single and dual target inhibitors based on Bcl-2: Promising anti-tumor agents for cancer therapy. Eur. J. Med. Chem..

[15] Zhong L., Liu Z., Yan R., Johnson S., Zhao Y., Fang X., Cao D. (2009). Aldo-keto reductase family 1 B10 protein detoxifies dietary and lipid-derived alpha, beta-unsaturated carbonyls at physiological levels. Biochem. Biophys. Res. Commun..

[16] DiStefano J.K., Davis B. (2019). Diagnostic and Prognostic Potential of AKR1B10 in Human Hepatocellular Carcinoma. Cancers.

[17] Li J., Guo Y., Duan L., Hu X., Zhang X., Hu J., Huang L., He R., Hu Z., Luo W. (2017). AKR1B10 promotes breast cancer cell migration and invasion via activation of ERK signaling. Oncotarget.

[18] Xie C., Ye X., Zeng L., Zeng X., Cao D. (2023). Serum AKR1B10 as an indicator of unfavorable survival of hepatocellular carcinoma. J. Gastroenterol..

[19] Zhong L., Shen H., Huang C., Jing H., Cao D. (2011). AKR1B10 induces cell resistance to daunorubicin and idarubicin by reducing C13 ketonic group. Toxicol. Appl. Pharmacol..

[20] Cassetta L., Pollard J.W. (2018). Targeting macrophages: Therapeutic approaches in cancer. Nat. Rev. Drug Discov..

[21] Helm O., Held-Feindt J., Grage-Griebenow E., Reiling N., Ungefroren H., Vogel I., Krüger U., Becker T., Ebsen M., Röcken C. (2014). Tumor-associated macrophages exhibit pro- and anti-inflammatory properties by which they impact on pancreatic tumorigenesis. Int. J. Cancer.

[22] Mantovani A., Allavena P., Marchesi F., Garlanda C. (2022). Macrophages as tools and targets in cancer therapy. Nat. Rev. Drug Discov..

[23] Bosco M.C. (2019). Macrophage polarization: Reaching across the aisle?. J. Allergy Clin. Immunol..

[24] Yunna C., Mengru H., Lei W., Weidong C. (2020). Macrophage M1/M2 polarization. Eur. J. Pharmacol..

[25] Gunassekaran G.R., Poongkavithai Vadevoo S.M., Baek M.C., Lee B. (2021). M1 macrophage exosomes engineered to foster M1 polarization and target the IL-4 receptor inhibit tumor growth by reprogramming tumor-associated macrophages into M1-like macrophages. Biomaterials.

[26] Comito G., Pons Segura C., Taddei M.L., Lanciotti M., Serni S., Morandi A., Chiarugi P., Giannoni E. (2017). Zoledronic acid impairs stromal reactivity by inhibiting M2-macrophages polarization and prostate cancer-associated fibroblasts. Oncotarget.

[27] Ilyas S.I., Khan S.A., Hallemeier C.L., Kelley R.K., Gores G.J. (2018). Cholangiocarcinoma–evolving concepts and therapeutic strategies. Nat. Rev. Clin. Oncol..

[28] DeOliveira M.L., Cunningham S.C., Cameron J.L., Kamangar F., Winter J.M., Lillemoe K.D., Choti M.A., Yeo C.J., Schulick R.D. (2007). Cholangiocarcinoma: Thirty-one-year experience with 564 patients at a single institution. Ann. Surg..

[29] Yan Y., Li Q., Shen L., Guo K., Zhou X. (2022). Chlorogenic acid improves glucose tolerance, lipid metabolism, inflammation and microbiota composition in diabetic db/db mice. Front. Endocrinol..

[30] Chang X., Monitto C.L., Demokan S., Kim M.S., Chang S.S., Zhong X., Califano J.A., Sidransky D. (2010). Identification of hypermethylated genes associated with cisplatin resistance in human cancers. Cancer Res..

[31] Hsu P.H., Chen W.H., Juan-Lu C., Hsieh S.C., Lin S.C., Mai R.T., Chen S.Y. (2021). Hesperidin and Chlorogenic Acid Synergistically Inhibit the Growth of Breast Cancer Cells via Estrogen Receptor/Mitochondrial Pathway. Life.

[32] Liu Y.J., Zhou C.Y., Qiu C.H., Lu X.M., Wang Y.T. (2013). Chlorogenic acid induced apoptosis and inhibition of proliferation in human acute promyelocytic leukemia HL-60 cells. Mol. Med. Rep..

[33] Olbromski P.J., Bogacz A., Bukowska M., Kamiński A., Moszyński R., Pawlik P., Szeliga A., Kotrych K., Czerny B. (2023). Analysis of the Polymorphisms and Expression Levels of the BCL2, BAX and c-MYC Genes in Patients with Ovarian Cancer. Int. J. Mol. Sci..

[34] Albalawi G.A., Albalawi M.Z., Alsubaie K.T., Albalawi A.Z., Elewa M.A.F., Hashem K.S., Al-Gayyar M.M.H. (2023). Curative effects of crocin in ulcerative colitis via modulating apoptosis and inflammation. Int. Immunopharmacol..

[35] Evan G.I., Vousden K.H. (2001). Proliferation, cell cycle and apoptosis in cancer. Nature.

[36] Wu C.C., Lee S., Malladi S., Chen M.D., Mastrandrea N.J., Zhang Z., Bratton S.B. (2016). The Apaf-1 apoptosome induces formation of caspase-9 homo- and heterodimers with distinct activities. Nat. Commun..

[37] Asadi M., Taghizadeh S., Kaviani E., Vakili O., Taheri-Anganeh M., Tahamtan M., Savardashtaki A. (2022). Caspase-3: Structure, function, and biotechnological aspects. Biotechnol. Appl. Biochem..

[38] Du W., Tu S., Zhang W., Zhang Y., Liu W., Xiong K., Zhou F., Li N., Zhang R., Yu J. (2024). UPP1 enhances bladder cancer progression and gemcitabine resistance through AKT. Int. J. Biol. Sci..

[39] Jiang W., Yang X., Shi K., Zhang Y., Shi X., Wang J., Wang Y., Chenyan A., Shan J., Wang Y. (2023). MAD2 activates IGF1R/PI3K/AKT pathway and promotes cholangiocarcinoma progression by interfering USP44/LIMA1 complex. Oncogene.

[40] Qu J., Li J., Zhang Y., He R., Liu X., Gong K., Duan L., Luo W., Hu Z., Wang G. (2021). AKR1B10 promotes breast cancer cell proliferation and migration via the PI3K/AKT/NF-κB signaling pathway. Cell Biosci..

[41] Jahchan N.S., Mujal A.M., Pollack J.L., Binnewies M., Sriram V., Reyno L., Krummel M.F. (2019). Tuning the Tumor Myeloid Microenvironment to Fight Cancer. Front. Immunol..

