# Supplementary figures and images for: Chlorogenic Acid as a Potential Therapeutic Agent for Cholangiocarcinoma
Source: Pharmaceuticals (Basel). 2024 Jun 17;17(6):794. doi: 10.3390/ph17060794 (PMC11206998; doi:10.3390/ph17060794)

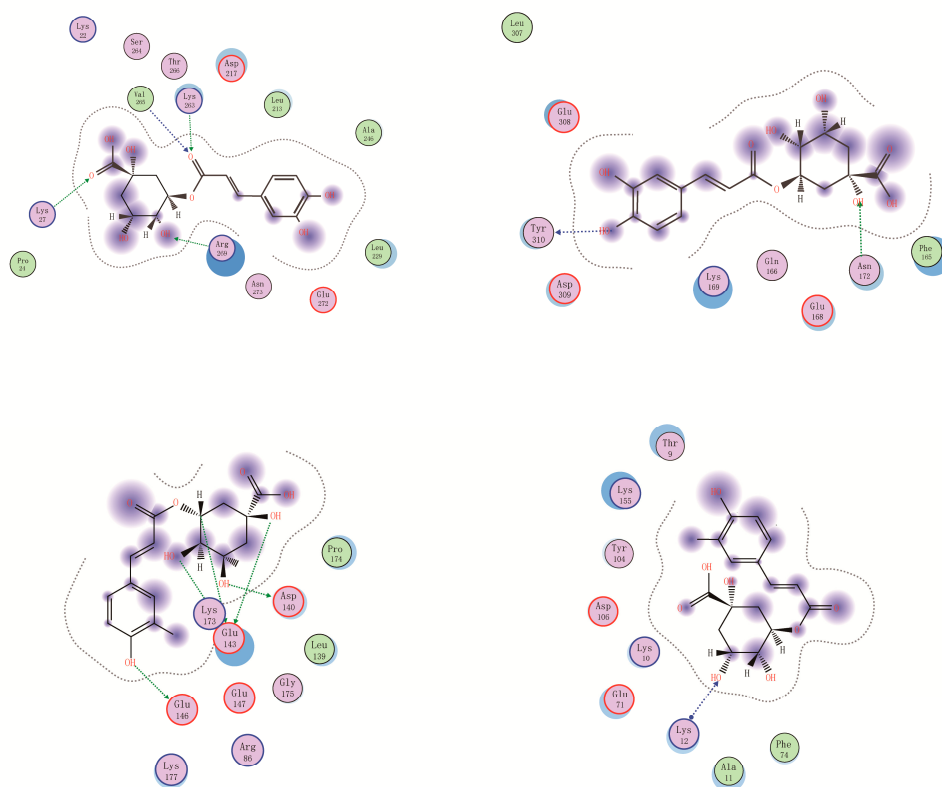

**Figure S1** Molecular docking results of chlorogenic acid and the protein of AKR1B10.

Supplement: Supplementary file 1 [file pharmaceuticals-17-00794-s001.zip › pharmaceuticals-3007827-supplementary.pdf]
